# FAMILY CAREGIVERS’ SELF-DIRECTED ENGAGEMENT WITH EVIDENCE-INFORMED CONTENT OF THE GAMEPLAN4CARE ONLINE PLATFORM

**DOI:** 10.1093/geroni/igae098.1846

**Published:** 2024-12-31

**Authors:** Alan Stevens, Jinmyoung Cho, Jordan Reese, Gang Han, Marcia Ory

**Affiliations:** Baylor Scott & White Health, Belton, Texas, United States; Saint Louis University School of Medicine, Ballwin, Missouri, United States; Baylor Scott & White Health, Belton, Texas, United States; Texas A&M University, College Station, Texas, United States; Texas A&M University, College Station, Texas, United States

## Abstract

GamePlan4Care (GP4C) translated one of the leading evidence-based dementia caregiving interventions, Resources for Enhancing Alzheimer’s Caregiver Health II (REACH II), into an online, interactive platform. Using decision algorithms with integrated online support from Dementia Care Specialists, GP4C includes the full breadth of REACH II education and skill-building materials tailored to the needs of the caregiver. The GP4C online system was provided to 120 family caregivers of persons living with dementia randomized to the GP4C arm of a two-arm clinical trial. The therapeutic content of GP4C included 7 domains: Safety, Stress, Health, Emotions, Care Services, Support, and Behaviors. Ninety-three family caregivers (77.5%) accessed the system. On average, caregivers accessed the platform for approximately 6 hours over 6 months and logged into the system an average of 14 times. Of the seven content domains represented in the system, Safety and Stress were the two most visited domains. Social Support and Health were the two least visited domains by caregivers. After 6-month use of the GP4C online platform system, caregivers reported a significant reduction in caregiver burden (effect size=.50; p<0.001) and a significant increase in the Positive Aspects of Caregiving measure (effect size=.36; p=0.002). Caregivers addressed several usability issues with the system and reflected that the content was more appropriate for those new to the role of dementia care. Modifications to the content and functionality of the GP4C system are ongoing and will be evaluated in future studies.

